# Groucho binds two conserved regions of LEF-1 for HDAC-dependent repression

**DOI:** 10.1186/1471-2407-9-159

**Published:** 2009-05-21

**Authors:** Laura Arce, Kira T Pate, Marian L Waterman

**Affiliations:** 1 Department of Microbiology and Molecular Genetics, University of California Irvine, 19182 Jamboree Blvd., Irvine, California USA, 92697-4025

## Abstract

**Background:**

*Drosophila *Groucho and its human Transducin-like-Enhancer of Split orthologs (TLEs) function as transcription co-repressors within the context of Wnt signaling, a pathway with strong links to cancer. The current model for how Groucho/TLE's modify Wnt signaling is by direct competition with β-catenin for LEF/TCF binding. The molecular events involved in this competitive interaction are not defined and the actions of Groucho/TLEs within the context of Wnt-linked cancer are unknown.

**Methods:**

We used *in vitro *protein interaction assays with the LEF/TCF family member LEF-1, and *in vivo *assays with Wnt reporter plasmids to define Groucho/TLE interaction and repressor function.

**Results:**

Mapping studies reveal that Groucho/TLE binds two regions in LEF-1. The primary site of recognition is a 20 amino acid region in the Context Dependent Regulatory domain. An auxiliary site is in the High Mobility Group DNA binding domain. Mutation of an eight amino acid sequence within the primary region (RFSHHMIP) results in a loss of Groucho action in a transient reporter assay. *Drosophila *Groucho, human TLE-1, and a truncated human TLE isoform Amino-enhancer-of-split (AES), work equivalently to repress LEF-1•β-catenin transcription in transient reporter assays, and these actions are sensitive to the HDAC inhibitor Trichostatin A. A survey of Groucho/TLE action in a panel of six colon cancer cell lines with elevated β-catenin shows that Groucho is not able to repress transcription in a subset of these cell lines.

**Conclusion:**

Our data shows that Groucho/TLE repression requires two sites of interaction in LEF-1 and that a central, conserved amino acid sequence within the primary region (F S/T/P/xx y I/L/V) is critical. Our data also reveals that AES opposes LEF-1 transcription activation and that both Groucho and AES repression require histone deacetylase activity suggesting multiple steps in Groucho competition with β-catenin. The variable ability of Groucho/TLE to oppose Wnt signaling in colon cancer cells suggests there may be defects in one or more of these steps.

## Background

**Figure 1:**
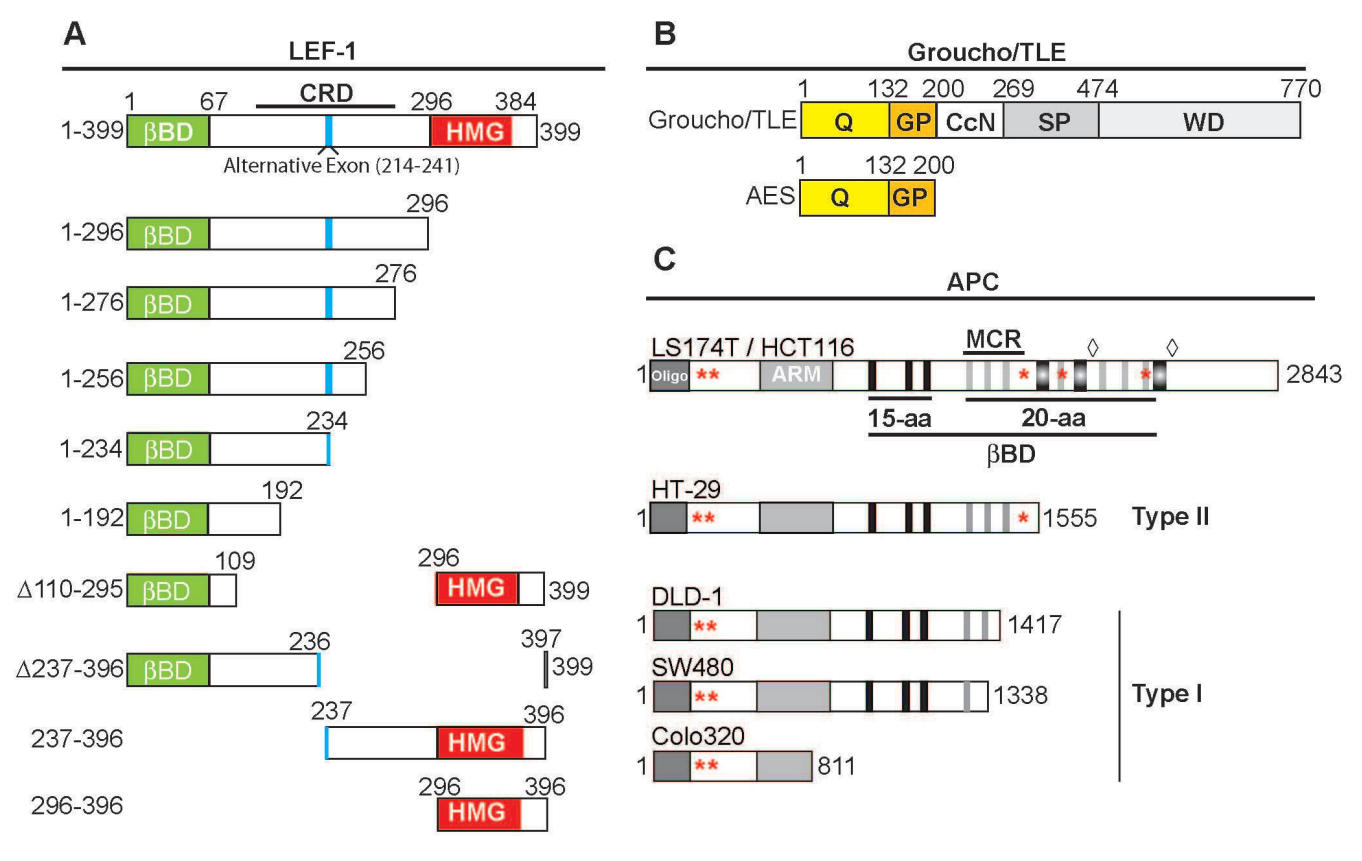
**Schematic representation of the LEF-1 and LEF-1 deletion constructs, Groucho and APC**. **A) **A LEF-1 protein schematic highlights four features: the β-catenin binding domain (βBD; aa1-67), the context dependent regulatory domain (CRD; aa67-296), an alternative exon in the CRD (black bar; aa214-241) and the high mobility group DNA binding domain (HMG; aa296-384). LEF-1 deletions tested in a GST pulldown assay are also depicted. **B) **General domain structure of Groucho/TLE proteins and truncated AES isoforms. The Q domain (aa1-132) is required for tetramerization and the GP domain interacts with HDACs. Three additional domains of protein-protein interaction are shown: the CcN, SP and WD-repeat domains. **C) **Schematic of Adenomatous polyposis coli (APC) and APC mutations found in Familial Adenomatous Polyposis (FAP) cancer cell of type I (DLD-1, SW480, and Colo 320) and type II (HT-29) and Hereditary Non-Polyposis Colon Cancer (HNPCC) colon cancer cell lines (LS174T and HCT116) [29,34]. Asterisks indicate nuclear export signals and diamonds indicate nuclear localization signals. The MCR (mutation cluster region) refers to the most common site of truncating mutations found in colon cancer. This region overlaps a region comprised of 15 and 20 amino acid repeats that bind beta-catenin. [28,48].

**Figure 2:**
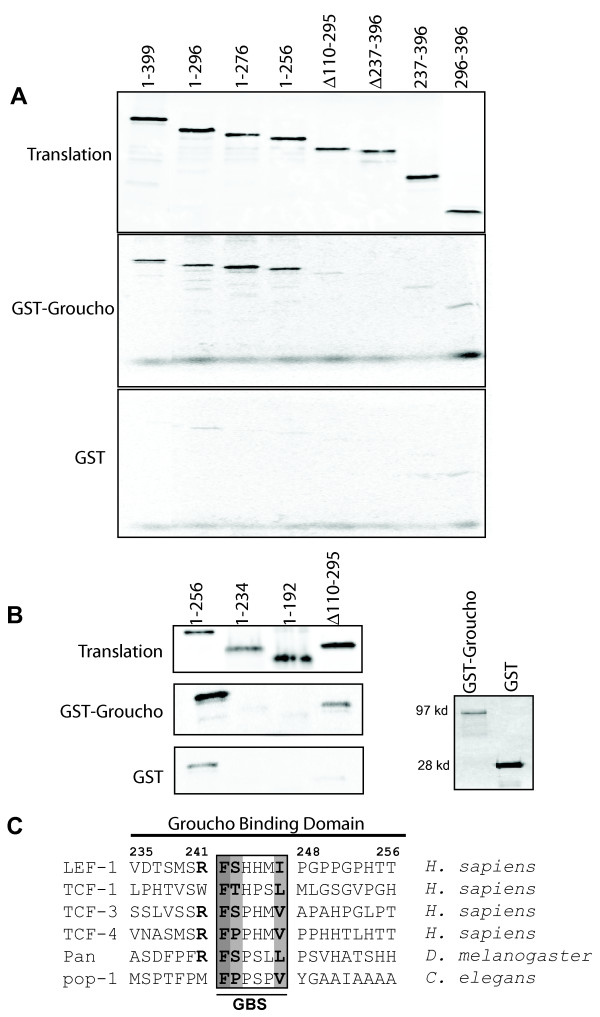
**Groucho binds to LEF-1 via a motif in the CRD and the HMG DNA binding domain**. **A) **Glutathione beads bound with either GST-Groucho (middle panel) or GST (bottom panel) were tested for interaction with *in vitro *translated ^35^S LEF-1 deletion constructs (translations shown in the top panel refer to deletions depicted in Fig. 1). **B) **Additional LEF-1 N-terminal deletions 1–234 and 1–191 (lanes 2 and 3) were not able to interact with GST-Groucho. Again, LEF-1 1–256 (lane 1) strongly interacts with Groucho but a deletion mutant lacking the entire CRD but retaining the HMG (Δ110–295, lane 4) weakly interacts. A GST negative control pull-down shows that LEF-1 does not interact with GST or the beads. Coomassie stained SDS-PAGE gel of purified GST-Groucho (lane 1) or GST alone (lane 2). **C) **Amino acid alignment of the putative Groucho binding sequence (GBS) and flanking sequences in LEF-1 and other human LEF/TCF family members as well as *D. melanogaster *and *C. elegans *worm orthologs, pangolin and POP-1 respectively. The LEF/TCF Groucho binding sequence described in this study, FS/T xxx I/L/V, is similar to the eh1 motif FxIxxIL [20]

The high mobility group (HMG) LEF/TCF transcription factors are well established activators and repressors that act to mediate Wnt signaling, but the details of how these proteins act on transcription are not complete. All LEF/TCFs are capable of recruiting the potent co-activator β-catenin via their N-terminal domain for activation (Figure 1A; [1,2]). Recruitment of β-catenin to target genes nucleates higher order activation complexes consisting of, in a partial listing, nucleosome modification and remodeling complexes, such as CBP/p300, SWI/SNF Brg1, Ada2/Ada3, TTRAP and MLL-I/SET-I [3-8]. In particular, β-catenin recruitment results in chromatin acetylation through the actions of recruited histone acetyltransferases (HATs). These recruited complexes positively regulate Wnt target genes including key cell cycle and cell proliferation genes.

LEF/TCF•β-catenin activation complexes are opposed by equally complex and multifaceted LEF/TCF•Groucho repressor complexes [9,10]. Groucho/TLEs are globally expressed, highly conserved WD-repeat proteins that function as transcription repressors [11,12]. Although Groucho/TLEs lack a DNA binding domain they are recruited to target gene promoters via direct interaction with transcription factors (bHLH, Runx, Pax5/BSAP, Six and LEF/TCFs) through one or more of their five protein interaction domains (Figure 1B; [12-16]). The C-terminal WD repeat domain in Groucho/TLE recognizes small amino acid motifs such as the well-studied pentapeptide WRPY/W motif through a beta-sheet propeller structure [17]. Proteins that contain WRPY/W-related motifs include the HES, Six3, Six2, NK-3 transcription factors and the Runx family of repressors [12,17-19]. Groucho/TLE can also recognize the eh1 peptide motif (**F**x**I**xx**IL**) originally described in the Engrailed (En) transcription factor, but also found in Pax, Six and other factors [14,20-22]. For LEF/TCFs, the Groucho binding domain has been assigned to the large central CRD region of the protein and the HMG DNA binding domain [23,24]. The CRD is approximately 130 amino acids in length, and is the most divergent region among the LEF/TCFs. The 88 amino acid HMG DNA binding domain is the most highly conserved region (Figure 1). Unlike the majority of identified Groucho interacting factors, no direct site of interaction or Groucho recognition motif has been found for LEF/TCFs. To better define Groucho/TLE interactions, we have delimited regions within LEF-1 that are important for Groucho binding. Our efforts have identified two interacting regions; the strongest is a sequence in the LEF-1 CRD (amino acids 241–248), and a second, weaker site of interaction in the LEF-1 HMG DNA binding domain (amino acids 296–396).

Groucho/TLE assembles a co-repressor complex with LEF/TCF factors to inhibit transcription of Wnt target genes. One component of the co-repressor complex is histone deacetylase-1 (HDAC1), and chromatin immunoprecipitation assays of LEF-1-based repressor complexes detect HDAC1 bound to Wnt target genes [5]. HDACs, which remove acetyl groups from chromatin to silence transcription, have been demonstrated to interact with Groucho/TLEs via the N-terminal GP-domain [25]. Since truncated Groucho members like Amino terminal Enhancer of Split (AES; Figure 1B) retain this region, we tested the hypothesis that both LEF•Groucho and LEF•AES complexes rely on HDAC activity in repression. Although some reports suggest that AES isoforms de-repress transcription, others have demonstrated that they provide repressive activities similar to full-length Groucho/TLEs [26,27]. Our findings show that like Groucho, the human AES family member uses HDAC activities to repress *in vivo*.

Just as there are many unknowns concerning Groucho interactions with LEF/TCFs, the dynamic switch back and forth between Groucho-based repression complexes and β-catenin-based activation complexes is not completely understood. The primary site of β-catenin interaction is the N-terminus of full-length LEF/TCFs, but an additional β-catenin interaction domain has been detected in the second half of the LEF-1 protein (CRD/HMG region) [24]. Discovery of this second site offers a new way to explain the observation that β-catenin and Groucho proteins exhibit mutually exclusive binding patterns in *in vitro *and *in vivo *binding assays. That is, it is proposed that the second β-catenin interaction in the CRD/HMG region might overlap a Groucho/TLE binding domain and compete for this site to destabilize interactions [24]. These *in vitro *observations suggest a model where LEF-1 is converted from an activator to a repressor by direct displacement of β-catenin by Groucho [24]. However, *in vivo *interactions suggest a more intricate switch. Sierra and colleagues have provided *in vivo *chromatin immunoprecipitation evidence that LEF-1 switches from an activator to a repressor in a multi-step process mediated by the transient association of the tumor suppressor Adenomatous Polyposis Coli (APC). Transient association of APC with the LEF-1•β-catenin complex occurs as β-catenin is released and before Groucho/TLE binding is detected [5]. Thus, displacement of β-catenin occurs prior to any Groucho association. Since the precise site of Groucho interaction has never been mapped, these *in vitro *and *in vivo *observations remain unresolved. That APC might be involved in the exchange between Groucho and β-catenin *in vivo *has importance for Wnt signaling and colon cancer where APC is either mutated or lost in these tumors, and it underscores how fundamental the unknowns are for Groucho interaction with the Wnt signaling pathway. Here we survey Groucho activity in six colon cancer cell lines that vary according to APC status and loss of β-catenin binding and nuclear localization motifs (Figure 1C, [28,29]). We find Groucho activity is compromised in half of these cell lines.

Groucho/TLE regulation is likely to have important effects on intestinal cell growth and differentiation but so far no loss-of-function mutations in TLEs have been reported. Relatively little is known about the role Groucho/TLE plays in Wnt signaling within the intestine, and it is especially understudied within the context of colon cancer. Our findings provide insight into the mechanisms of Groucho/TLE mediated repression of the Wnt pathway and suggest that APC status in colon cancers may play a role in the dynamic interplay between the high levels of β-catenin and the ubiquitous Groucho/TLE proteins and ultimately the lack of repression in cancer.

## Methods

### Cell lines/Transfections

Colon cancer cell lines, SW480, Colo320, DLD-1, LS174T and the monkey kidney cells, COS-1 were cultured in either Dulbecco's modified Eagle's medium (DMEM) or RPMI-1640 medium, supplemented with 10% Fetal Bovine Serum (FBS), at 37°C and 5% CO_2_. COS-1 cells were transfected with FuGENE 6 (Roche) and cancer cell lines were transfected with Effectene (Quiagen). For HDAC inhibition, cells were treated with Trichostatin A (TSA) (50 ng/mL) for 24 hours.

### Plasmids

Mammalian expression and reporter vectors were generously provided by Dr. Hans Clevers, Hubrecht Laboratory, Netherlands Institute of Developmental Biology (TOPFlash and Groucho), by Dr. Randall Moon, University of Washington (SuperTOPflash), and by Dr. Takashi Okamoto Department of Molecular Genetics, Nagoya City University Medical School (mammalian expression vector pCMV His-AES). and Dr. Wayne Phillips, Peter MacCallum Cancer Center, Melbourne, Australia (human TLE-2 expression vector). The full length human LEF-1 coding sequence cloned between BamHI and NheI in pEV3S includes 285 bp of the 3' untranslated region of LEF-1 and is the backbone to all of the LEF-1 deletion constructs (see also [30]). Bacterial expression of both the GST and GST fusion proteins were generated with the pGEX expression vectors. To construct the GST-Groucho expression vector, Groucho (*Drosophila*) coding sequences were excised from pCDNA3 using BstX I and the overhangs filled by a Klenow reaction. The fragment was inserted in-frame into pGEX3X linearized with SmaI. The construct was sequenced to verify frame. The LEF-1 Groucho binding sequence substitution mutant (GBS; 241/248) and downstream substitution mutant 250/255 were generated via site-directed mutagenesis (Stratagene Protocol). The LEF-1 GBS (mutation of AA 241–248) was created using the primer sequence 5'-acttccatgtccggggtagcgctagcactagcagccggtcctcctggtccc-3'. The LEF-1 250/255 mutation was created using the primer sequence 5'-catatgattcccggtgtagcgctagcactagcaactggcatccctcat-3'.

### Protein-Protein interaction Assay

Recombinant GST or GST-Groucho was produced in *Escherichia coli *strain BL21 and recombinant protein purified over a GST column (Amersham). Fractions were eluted, and dialyzed (20 mM Tris-HCl (pH7.9), 1 mMDTT, 10 mM MgCl2, 0.05% Nonidet P-40, 10% glycerol). LEF-1 and LEF-1 deletion constructs were ^35^S methionine labeled with the TNT *in vitro *transcription/translation system (Promega). GST-tagged proteins were immobilized on Glutathione sepharose beads (Amersham) for 1 hour at 4°C in lysis buffer (200 mM NaCl, 0.5% Tween 20, 10 mM Tris-Cl and a cocktail of protease inhibitors). Immobilized protein was incubated with ^35^S-LEF-1 labeled proteins in binding buffer (200 mM NaCl, 0.5% NP40, 10 mM Tris-Cl, 5 mM MgCl2 and 0.2% BSA) for 30 minutes at room temperature. Beads were washed 3 times for 10 minutes at room temperature and then resuspended in 15 uL of 2× SDS Sample buffer. Eluted proteins were separated on a 15% SDS- PAGE. For visualization, gels were incubated for 30 minutes at room temperature in EN3Hance solution (Perkin Elmer), washed for 5 minutes in distilled water, dried, and exposed to film.

### Immunoblotting

For COS-1 cell extracts, COS-1 cultures were transiently transfected and harvested 48 hours post-transfection. Cells were resuspended in 2× SDS sample buffer. For western analysis of TLE expression in colon cancer cells, extracts were prepared with lysis buffer (50 mM Tris-HCl, 120 mM NaCl, 0.5% NP-40, 8 mM MgCl_2_, 1 mM DTT, protease inhibitor cocktail), added to 2× SDS sample buffer, and balanced to load 35 μg total protein onto the protein gel. Extracts were separated by SDS-PAGE and transferred to a nitrocellulose membrane. The transfer membrane was blocked with 5% milk TTBS (TBS, 20 mM Tris, 500 mM NaCl, pH 7.5 TTBS, TBS, 0.05% Tween-20, pH 7.5) solution at room temperature for 30 minutes and incubated overnight at 4°C with primary antibody (Anti-His antibody (1:250, Santa Cruz Biotenology), Anti-Myc antibody (1:1000, Santa Cruz Biotechnology), or Anti-panTLE rat monoclonal antibody (1:1000) [31]) were diluted in 1% milk TTBS). The membrane was washed 3 times for 10 minutes in TTBS, followed by incubation with secondary antibodies (anti-mouse or rabbit IgG antibodies (1:5000, Amersham) or anti-rat IgG (1:50,000)) for 2 hours at room temperature. Blots were washed 3 times in TTBS and developed using ECL kit (Amersham) and exposed to film.

## Results

### LEF-1 contains two Groucho binding domains

All LEF/TCFs interact with Groucho/TLE family members, but a specific site of interaction has yet to be defined [24,27]. We delimited the sequences important for Groucho binding using an *in vitro *protein interaction assay with purified recombinant GST-Groucho protein (*Drosophila*) and LEF-1 deletion proteins produced via *in vitro *translation in the presence of ^35^S-methionine (Figure 2). The expected size of recombinant GST-Groucho is 125 kDa, but we recover a 97 kDa protein product (Figure 2B). Therefore, purification of GST-Groucho results in a protease stable fusion protein lacking part of the C-terminal WD-repeat domain but retaining more N-terminal domains including the Q and GP domains. Since the N-terminal Q domain has previously been shown to be sufficient for interaction with LEF/TCFs, we proceeded with this GST-Groucho preparation for a finer mapping of the recognition region within LEF-1 [24,27]. Schematic representations of the LEF-1 deletion mutants tested in the pull-down assay are shown in Figure 1A. Full length LEF-1 (1–399) as well as LEF-1 C-terminal deletions, retaining either the entire CRD (1–296) or N-terminal portions of the CRD (1–276 and 1–256), are all able to specifically interact with GST-Groucho (Figure 2A, middle panel, lanes 1–4). However, a LEF-1 internal deletion lacking the C-terminal half of the CRD and the entire HMG DNA binding domain (Δ237–396) does not interact with GST-Groucho (lane 6). In addition, an internal deletion removing most of the CRD but retaining the HMG DNA binding domain, is weaker in its ability to bind GST-Groucho (Figure 2A, lane 5; Figure 2B, lane 4; Δ110–295). A LEF-1 fragment containing the C-terminal half of the CRD and the entire HMG domain (237–396) or a fragment consisting of only the HMG DNA binding domain (296–396) are equally modest in their ability to bind Groucho (lanes 7 and 8). Groucho binding is lost only when both the CRD and the HMG DBD are deleted (Δ237–396) (lane 6). Taken together, these data suggest there are two areas of interaction. The primary site is located in the CRD upstream of amino acid 256 and a second, weaker site of interaction is in the HMG DNA binding region between residues 296 and 396. To identify residues most important for binding, we tested additional deletions of the strongest site of interaction in the CRD. C-terminal deletion to amino acids 234 or 192 abrogated binding to GST-Groucho thus delimiting a 20 amino acid region of recognition: 237-TSMSRFSHHMIPGPPGPHTT-256 (Figure 2B, C). While this region contains no obvious matches to the well-known WRPW/Y motif recognized by the Groucho WD-repeat domain, it shares weak similarity to the eh1 binding motif (**F**x**I**xx**IL**) in that a phenylalanine residue is present 4 and 5 residues away from two bulky hydrophobic residues [17,20]. Eh1 motifs are often recognized by the WD-repeat domain of Groucho/TLE, but a subset are recognized by the Q-domain (see Discussion). Weak similarities to eh1 via the presence of hydrophic residues in the middle of this 20 amino acid region does not definitively assign a small recognition motif, but we nevertheless hypothesize the main site of recognition to be **F S/T/P **xx y **I/L/V **where x is a histidine, proline or serine (Figure 2C). Even though the entire CRD is poorly conserved among LEF/TCF family members and orthologs, this central hydrophobic sequence is conserved in mammalian, fly and worm TCFs suggesting that all LEF/TCFs might be recognized by Groucho in their respective CRDs through this sequence which we refer to hereafter as the Groucho Binding Sequence or GBS (Figure 2C).

### Groucho and hAES repress LEF-1 mediated activation *in vivo*

**Figure 3:**
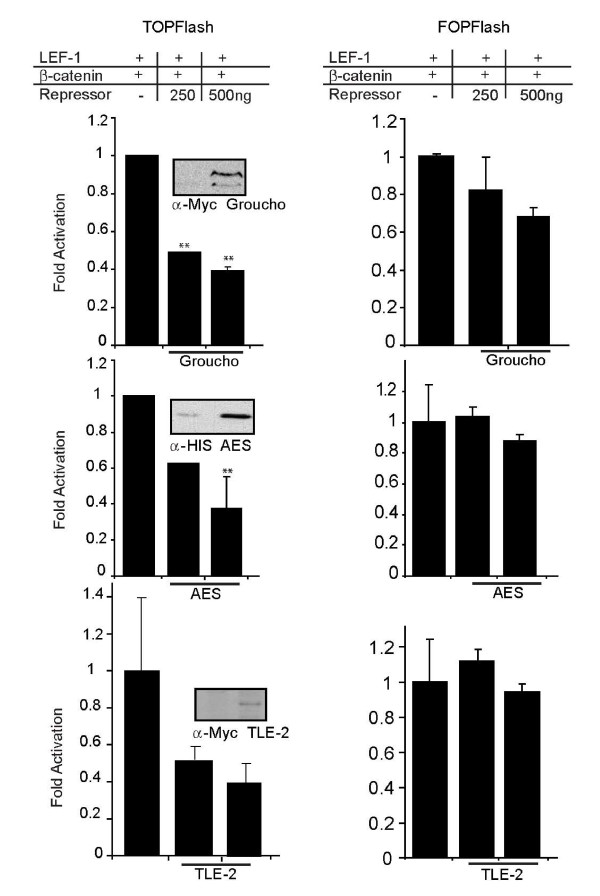
**Groucho and AES repress LEF-1/β-catenin mediated activation of transcription**. Luciferase activity of a transiently transfected TOPFlash reporter assay using COS-1 cells. The TOPFlash (100 ng) reporter with three multimerized LEF/TCF DNA consensus sequences in front of the c-fos minimal promoter was activated 5 and 3-fold by LEF-1•β-catenin complexes (200 and 400 ng of expression plasmid respectively). This level of activation was set equal to one for comparison to the amount of TOPflash activity measured in the presence of Groucho or AES expression vector (250 and 500 ng as shown). Increasing amounts of Groucho or AES expression vector cause increasing repression of reporter activity. All data was normalized with a co-transfected CMV β-galactosidase reporter. Error bars represent the standard deviation among three experiments. Student t test was performed and p values are represented by asterisks. One asterisk represents a value < 0.05 and two asterisks represent a value of < 0.01. Western analysis of Groucho, AES and TLE-2 expression shows a dose-dependent increase in protein expression (insets).

Typically, Groucho interacts with transcription factors via its WD-repeat domain, but in the case of LEF/TCFs, binding occurs via the Q domain [24,27]. The adjacent GP domain, which is known to recruit histone deacetylases (HDACs), is present in truncated Groucho family members such as AES (Figure 1; [24,25]). These isoforms contain only the N-terminal Q and GP domains and have reported actions that vary between either de-repression (mGrg-5 and XGrg-5) and repression (hAES) [23,26,27]. The differences in repressor or de-repressor function seem to correlate with sequence differences in the GP domain and may therefore be the basis for the opposite actions of the very different mAES and XGrg-5 family members [27]. We compared the ability of LEF-1 to recruit full-length *Drosophila *Groucho, human TLE-2 and human AES for transcription regulation (Figure 3). Using the TopFlash reporter plasmid, which contains three multimerized Wnt response elements, we activated transcription by transiently co-transfecting LEF-1 and β-catenin expression vectors in COS-1 cells. Activation by LEF-1•β-catenin complexes was equivalently repressed by Groucho, hTLE-2 and hAES co-repressors two- to three-fold (Figure 3, left column). Western analysis of transiently transfected Myc-tagged Groucho, Myc-tagged hTLE-2 or His-tagged hAES expression vectors in COS-1 cells was performed to confirm expression (Figure 3, panel insets). Transcription repression was specific for each as TOPFlash activity was reduced whereas there were no significant changes in activity produced from the negative control FOPFlash, a reporter that contains 3 multimerized mutant LEF/TCF binding sites (Figure 3, right column). A co-transfection control plasmid expressing LacZ is also unaffected by these conditions. It is notable that *Drosophila *Groucho is an equally effective co-repressor for hLEF-1 as human TLE-2 and human AES, an action that must be functionally conserved across species [23,27]. Given our findings that hAES acts as a co-repressor for LEF-1, we conclude that recognition of LEF-1 via the Q domain is sufficient for repression of β-catenin actions and using this validated assay we then tested whether the delimited region in the CRD is able to recruit Groucho for transcription repression *in vivo*.

### Mutation of the Groucho Binding Sequence impairs repression *in vivo*

**Figure 4:**
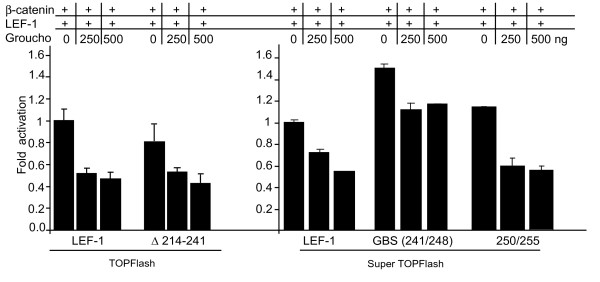
**The Groucho Binding Motif in LEF-1 is necessary for repression**. Luciferase activity in transiently transfected COS-1 cells. The TOPFlash or Super TOPFlash reporters were co-transfected with the indicated LEF-1 deletion constructs (200 ng) and β-catenin (400 ng) with increasing amounts of Groucho expression vector (indicated). Repression is maintained when activated by LEF-1 lacking the alternative exon (Δ214–241; left panel). Repression is also maintained when residues next to the GBM are changed (250/255). Repression is attenuated when all eight residues of the GBM are altered (right panel, center). Error bars represent the spread between duplicates and is representative of more than four replicate experiments.

LEF-1 amino acid substitutions of the GBS were tested for their potential to disrupt Groucho action in cells. We first tested a deletion (Δ214–241) which removes alternatively spliced exon sequences in the CRD (see Figure 1A), sequences juxtaposed N-terminal to the GBS. This region has been identified as a HIC5 repressor interaction site and we wished to test whether the absence of this domain affected the ability of Groucho to repress [32]. We observed that deletion of these residues had no effect. Groucho repression levels were similar to levels with full length, wildtype LEF-1 (Figure 4, left panel). Thus, alternative splicing in the upstream region does not affect Groucho/TLE repression in our assay. However, when site directed mutagenesis was used to change the eight central amino acids that includes the delimited Groucho Binding Sequence (241–248; RFSHHMIP to GVALALAA), much of the Groucho mediated repression was lost (Figure 4, right panel). Substitution of the neighbouring six residues (250/255) had no effect on Groucho repression. These *in vivo *data are functional confirmation that amino acids RFSHHMIP (241–248) encompass the core Groucho binding site.

### Groucho mediated repression is HDAC dependent

**Figure 5:**
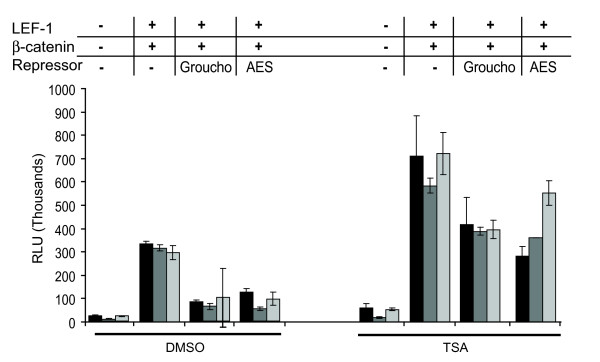
**Groucho and AES recruit HDAC for transcription repression**. LEF-1•β-catenin activated TOPFlash was repressed by Groucho or AES (1000 ng) via transient transfection in COS-1 cells. Treatment with the HDAC inhibitor trichostatin A (50 ng/ml) relieved repression. Data is reported as described in Figure 3. Three independent experiments are shown. The error bars represent the spread of the duplicates for each experiment.

Groucho/TLE repressors are transcription regulators that interact with many other co-regulatory factors including the chromatin modifier HDAC (histone deacetylase), an interaction that is functionally conserved from flies to humans [33]. To determine if LEF-1•Groucho complexes require HDAC activity for the repression observed in our assay, we tested whether the HDAC inhibitor trichostatin A (TSA) could relieve Groucho and/or AES-mediated repression. The results in Figure 5 show that indeed, TSA interfered with Groucho and AES repression. TOPFlash activity was repressed by both Groucho and AES 4–5-fold in the presence of DMSO. However, reporter activity was not very well repressed by Groucho or AES in TSA treated cells (Figure 5; 1.7-fold). Although β-catenin activation of TOPflash was increased 2.1-fold in the presence of TSA, this was also true of reporter basal activity which improved 2.5-fold, suggesting that the core promoter in this vector is influenced by the actions of HDACs and β-catenin function is unaffected. These data provide evidence that the transient transfection assay detects Groucho mediated repression through HDAC-dependent actions. That TSA effects were similar for full-length Groucho and the shorter AES isoform is consistent with previous reports that HDACs are recruited by the GP domain.

### Groucho represses β-catenin action in APC class I mutant colon cancer cells

**Figure 6:**
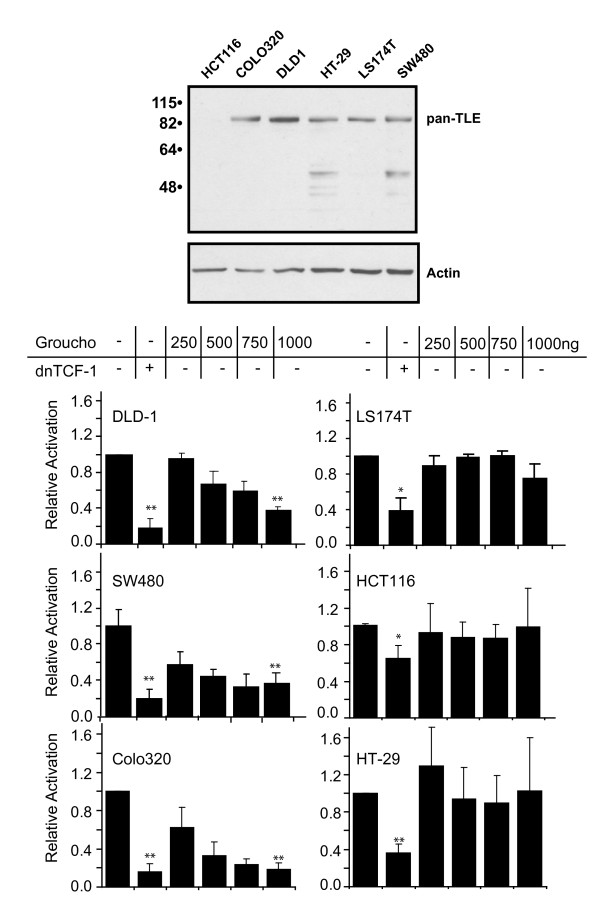
**Groucho action in colon carcinoma derived cells**. **A) **Western analysis of endogenous TLE expression in colon cancer cell lines. Whole cell extracts from the indicated colon cancer cell lines were analyzed by western blotting. Blots were probed with rat monoclonal antisera with pan specificity for the TLE family of proteins. Longer exposure of the blot reveals low levels of TLE protein in HCT-116 cell line (data not shown). **B) **Groucho activity was assessed in colon cancer cell lines. SW480, Colo320 and DLD-1 cells are type I APC mutant cells (left panels). LS174T, HCT116 and HT-29 cells are wild type APC or type II APC mutant cells (right panels). Refer to Figure 1 for the corresponding APC mutations in these cells. TOPFlash reporter plasmid was co-transfected with either a dominant negative TCF-1 expression vector (400 ng) or increasing amounts of Groucho expression vector. Luciferase activity was assayed 24 hours post transfection and activities normalized to TOPFlash reporter alone. The error bars show the standard deviation derived from three independent experiments.

If Groucho uses a co-recruited HDAC activity to oppose β-catenin•LEF-1, is this action sufficient to counter endogenous β-catenin and its recruited histone acetyltransferase complexes in colon cancer cells? As previously mentioned, a recent study in colon cancer cells suggests that the switch between β-catenin and Groucho/TLE is complex and is mediated by the tumor suppressor APC [5]. The most common mutations in colon cancer and derived cell lines result in truncating mutations of the APC protein. Truncated APC proteins lead to the stabilization of β-catenin and overactive Wnt signaling because the cytoplasmic destruction complex cannot effectively capture and degrade β-catenin. Elevated levels of β-catenin in the nucleus might be predicted to influence the dynamics of a competition with Groucho proteins for binding to endogenous LEF/TCFs in cells. However, the ability of Groucho overexpression to counteract high levels of β-catenin in colon cancer has never been compared between colon cancer cell lines. We surveyed Groucho action in a set of six different lines that differ with respect to mutations in the APC and β-catenin loci. All cells have higher than normal levels of LEF/TCF•β-catenin complexes in the nucleus due to either stabilizing mutations in β-catenin or loss-of-function mutations in APC. Type II cell lines (HT-29 cells) harbour truncating mutations in APC that produce a shortened APC lacking two nuclear export and nuclear localization signals (NES; Figure 1) [29,34]. Type I cell lines (DLD-1, SW480 and Colo320 cells) harbour mutations that severely truncate APC and lack all but two NESs (Figure 1). The third type (HCT116, LS174T) contains wildtype APC, but has stabilizing mutations in β-catenin [35,36]. TLE expression in colon cancer cell lines has been analyzed by RT-PCR and multiple TLE family members are expressed in each of these cell lines [27]. Despite the detection of TLE mRNAs, variations in the levels of TLE protein could be an important factor in β-catenin activity in the nucleus. Since the levels and patterns of TLE protein have never been reported we used whole cell extracts and a rat monoclonal antisera with pan-TLE specificity to assess TLE expression by Western blotting [31]. Figure 6A shows that TLE expression is similar in five of the six cell lines. DLD1 cells appear to have higher levels of TLE but the HCT116 cell line has very low levels (longer exposure of the western blot shows that TLE protein is detected, data not shown). Therefore at least for HCT116 cells, β-catenin is in much greater excess relative to total TLE protein.

Each of these cell lines were transiently co-transfected with the TOPFlash reporter and increasing amounts of Groucho expression plasmid. For comparison, TOPFlash was co-transfected with an expression vector for dominant negative TCF-1 (dnTCF-1) which competes with endogenous LEF/TCF•β-catenin complexes for binding to the reporter. We observed significant repression in all of the type I APC cell lines similar to the negative effects of dnTCF-1. For example at maximal levels of repression by Groucho, TOPFlash activity was reduced 3-fold in DLD-1 cells, 2.5-fold in SW480 cells and 5-fold in Colo320 cells (Figure 6, left). Surprisingly, there was no significant Groucho repression in APC type II, HT-29 or the APC wild type, β-catenin mutant, LS174T and HCT116 colon cancer cells (Figure 6, right). These data suggest that colon cancers may differ in the dynamic interplay between β-catenin and Groucho/TLE such that β-catenin is less opposed in its actions in some colon cancers and not others.

## Discussion

### Two interaction domains for Groucho and LEF-1

Groucho/TLEs are described as the main transcription repressor that opposes Wnt target gene activation, but the molecular details of their interaction with LEF/TCFs are not well understood [37]. Our data define a direct interaction between Groucho and LEF-1 as occurring via two regions, a small region in the context regulatory domain (aa237-256) and a region in the highly conserved HMG DNA binding domain (aa296-396). It is not unprecedented for Groucho to interact with two regions of a transcription factor. For example, Groucho binds to two different domains of PAX5 protein via its SP and Q domains, and to [14,18]. Groucho/TLE functions as a tetramer and therefore a multimer of Groucho/TLE could occupy both LEF-1 regions simultaneously (although our assay could also be detecting independent binding to separate LEF-1 protein molecules). Chen et al. have shown that Groucho tetramers can form higher order multimers with other Groucho tetramers, and Daniels and Weis demonstrated that recombinant TLE Q fragment indeed forms tetramers *in vitro *[24,38]. Thus, these higher order complexes could easily span the two domains of LEF-1 for simultaneous binding. In fact, Daniels and Weis have calculated a 4:1 molar ratio for TLE-1/LEF-1 complexes [24].

### HMG interactions

The interaction between Groucho and the HMG region may be indicative of a general feature of HMG domains since the two HMG boxes of HMGB1 interact with mouse Grg1 and Grg5 [39]. The interaction we define between the LEF1 HMG and Groucho is direct via protein/protein interaction and not mediated by artifactual DNA tethering since the pulldown assays were performed in the presence of the DNA intercalating agent Ethidium Bromide. Our results indicate that the HMG interaction is weak and not sufficient for Groucho/TLE repression *in vivo*, because a deletion mutation that removes the CRD but retains the HMG DNA binding domain does not support Groucho-mediated repression (data not shown). Therefore, Groucho requires the CRD for action *in vivo*. However, we also tested whether the CRD alone was able to recruit Groucho/TLE for repression. In this case we fused LEF1 coding sequences for aa1-256, 1–276- and 1–296 to the yeast Gal4 DNA binding domain and co-transfected these expression vectors with a reporter plasmid containing 5 multimerized Gal4 binding sites. While we observed Groucho-mediated repression, the effects were much more variable than with HMG-based proteins (data not shown). Therefore an interaction with the CRD may be the main functional site for Groucho binding, but auxiliary interactions with the HMG DNA binding domain might further stabilize the complex.

### GBS interactions

Our *in vitro *assays were designed to focus on interactions within the LEF-1 context dependent regulatory domain, and a 20 amino acid region responsible for much of the Groucho binding was delimited. Mutation of the central 8 residues in this region eliminated Groucho-mediated repression *in vivo *(241–248; Figure 4, right panel). Six of the eight residues comprise a conserved sequence among orthologs and family members and we therefore propose that these 6 residues are the primary site of Groucho binding and recognition. This sequence, or Groucho Binding Sequence (GBS) 241-FSHHMI-248, is short like most Groucho interaction motifs, most notably eh1-motifs (**F**x**I**xx**IL**) and W/VRPY motifs, in that aromatic and bulky hydrophobic residues are important. All LEF/TCFs contain a similar GBS-like sequence, and in fact, the analogous sequences in TCF-3 and TCF-4 (Figure 2; **FSP**HM**VA**, **FPP**HM**VP **respectively) are comparable to Groucho/TLE binding motifs in mSix3 and mSix6 transcription factors (**FSP**EQ**VA**, **FSP**QQ**VA **respectively) [22]. Similar to what has been determined for LEF-1, it is the Q domain that binds to the Six motifs [18]. It is possible that the GBS is a variant of eh1, but there are important characteristics that distinguish it from this class of motif. For example, while our mutation data show the GBS to be necessary for Groucho action, an eight amino acid synthetic peptide containing the GBS was unable to compete with *in vitro *translated LEF-1 proteins for GST-Groucho binding, even when the peptide was provided at high molar excess. In contrast, eh1 motifs can independently bind to Groucho either as a purified, synthetic peptide of six amino acids or as a small fusion to the C-terminus of GST [17]. Either our synthetic GBS peptide was unable to fold into the appropriate conformation or neighbouring residues are involved in recognition. A requirement for neighbouring residues may be indeed be important and may be the reason why LEF-1 fragment 237–396 does not bind very well to Groucho in the *in vitro *GST-pulldown assay (Figure 2, lane 7). The GBS is present in this protein fragment, but is flanked at its N-terminus by only three residues. Protease protection studies with GST-TLE-1 and LEF-1 show that Groucho binding protects a region of LEF-1 starting at amino acid 216, a full 25 residues upstream of the GBS [24]. It is possible that unlike eh1 and WRPW motifs, an extended interaction is important for stable binding.

There are other distinguishing characteristics between the GBS and eh1 motfs. Eh1 peptides adopt alpha helical conformations, particularly when bound to the WD-repeat domain of Groucho [17]. The secondary structure of our sequence is unknown, but a conserved alpha helical conformation is unlikely as the TCFs and other LEF/TCF orthologs have helix-breaking proline residues in the middle of the motif. Also, unlike eh1, it is the Q domain of Groucho, not the WD repeat domain that is important for binding.

### β-catenin and Groucho competition and HDAC requirements

An elegant biochemical and structural study by Daniels and Weis showed that in addition to binding the N-terminus of LEF-1, β-catenin binds to a second region that spans the latter half of the protein (252–397) including the HMG DNA binding domain [24]. They also showed in this study that Groucho/TLE binding protects a large region of LEF-1 from aa216 through aa397. Based on these observations, a model was proposed in which the overlapping β-catenin•HMG interactions competitively displace Groucho/TLE from LEF-1 for Wnt target gene activation. Our data shows that Groucho has a primary binding site of interaction in the CRD (GBS: aa241-248) distinct from the β-catenin secondary site (aa252-397), but a site that is closely juxtaposed. Also, the Groucho auxiliary site lies squarely within the same HMG DNA binding region protected by β-catenin (aa296-396). Thus, the competitive displacement model proposed by Daniels and Weis could be due to steric hindrance of either the primary or secondary site, or interference with both [24].

Our transient transfection assay, in which both co-activator β-catenin and co-repressor Groucho are overexpressed, is primarily an assay for competitive displacement. Therefore it was surprising that addition of the HDAC inhibitor TSA caused a significant reduction in the ability of Groucho and AES to compete with β-catenin and repress TOPflash expression. Could TSA be destabilizing Groucho competition for LEF/TCF binding? Groucho/TLE/Grg proteins bind directly to histone tails but not if these tails are acetylated [33]. This binding ability has been shown to be important for recruitment and interaction with chromatin-bound transcription factors FoxA1 and Hes1 [40]. Stable Groucho association with these factors was detected only when chromatin was unacetylated. Groucho also did not stably associate when the factors were bound to naked DNA, or when templates were assembled with tail-less histone H3 and H4 nucleosomes, or when assembled with highly acetylated chromatin [40]. The exact nature of chromatin assembled on transiently transfected plasmids is unknown, but partial chromatinization and semi-regular arrays of nucleosomes are likely to be present [41-43]. Perhaps in our system the TSA-sensitive HDAC activity is essential for deacetylating chromatin to enable stabilized Groucho interactions with DNA-bound LEF-1 and neighboring nucleosomes. Stabilized interactions might be necessary for Groucho to effectively displace β-catenin. Since one of the hallmarks of β-catenin action is chromatin acetylation, we favor a model that without co-recruited HDAC activity, Groucho binding to DNA-bound, β-catenin-occupied LEF/TCFs is tenuous and transient and β-catenin wins the competition for LEF-1 occupancy. Groucho/TLE proteins have additional maintenance actions on chromatin and transcription such as nucleosome-based spreading and chromatin compaction, but it is not very likely that our transfection assay is assessing these more long-term activities.

### Variations in Groucho activity in colon cancer cell lines

Groucho suppression of Wnt signaling would be reduced if its binding to LEF/TCFs were impaired, or as our data show, its ability to utilize HDAC activity were prevented. Many cancers have elevated Wnt signaling, but so far, no mutations in the CRD of any LEF/TCF have been reported and no mutations in Groucho/TLE or HDACs have ever been described. Instead, the majority of colon cancers harbour truncating mutations in APC or stabilizing mutations in β-catenin. Mutations in APC might have important bearing on Groucho action because at least one recent study has shown that APC can facilitate displacement of β-catenin and association of TLE-1 at an endogenous Wnt target gene [5]. In this study, HT-29 colon cancer cells with truncated APC have LEF-1/β-catenin complexes constitutively occupying a WRE in the cMYC enhancer. When wildtype, full-length APC was inducibly expressed in these cells, nuclear localization and association of the overexpressed wildtype APC with the chromatin-bound LEF-1 complex was detected [5]. This association with LEF-1 was transient and preceded rapid displacement and exchange of β-catenin for TLE-1 [5]. Based on these observations a two-step model was proposed whereby APC mediates the competition and exchange between the co-activator β-catenin and the co-repressor Groucho/TLE for LEF/TCF binding.

To further investigate the role of APC in repression, we tested the ability of Groucho to repress TOPFlash activity in colon cancer cell lines with wild type or variant APC proteins, predicting that Groucho would not be able to oppose β-catenin actions in cell lines with truncated APC proteins. Surprisingly, our data suggests the opposite. Groucho activity is most evident in cell lines with truncated APC protein. While the underlying mechanism for this is unknown, a survey of the literature reveals that APC localization differs in colon cancer cells lines but follows a trend based on APC length. Specifically, the HT-29 colon cancer cell line has a type II APC truncation mutation and the LS174T and HCT116 cell lines have a full length, wild type APC protein [34]. Each of these cell lines have predominantly cytoplasmic APC [29]. Conversely, DLD-1, SW480 and Colo320 colon cancer lines have high levels of nuclear APC; all of these lines have type I mutations that result in significantly truncated APC protein [44-47]. Interestingly, the type II and wild type APC proteins retain three of five NES sequences and the type I APC mutations retain only two NES sequences (Figure 1). Although both NLS sequences are located in the C-terminus, mutants lacking this region are still localized in the nucleus, a localization that might be due to its association with nuclear β-catenin [29].

Our results suggest that nuclear APC predicts effective Groucho/TLE repression. Thus, the observation in HT-29 cells where induced expression of wildtype APC clears β-catenin from Wnt Response Elements could be due to the fact that overexpression of APC forces greater levels of its nuclear concentration [5]. Our data also point to additional abnormalities. HCT116 cells have very low levels of total TLE protein, as determined by western blot with pan-TLE-specific antibody (Figure 6A). These low levels may be due to TLE protein instability because TLE mRNA is present [27]. We transiently overexpressed hTLE2 in these cells as a positive control but were unable to detect hTLE2 protein on a western blot (data not shown). Therefore the lack of Groucho action in HCT116 cells might be due to the fact that TLE proteins are unstable in this cell line. This unexpected observation suggests that TLE protein levels and stability may vary in colon tumors and cell lines.

## Conclusion

We conclude that Groucho/TLE repression of β-catenin association and action with LEF/TCFs requires two sites of interaction. The primary site of Groucho interaction is a short conserved amino acid sequence in the context dependent regulatory domain. Repression requires histone deacetylase activity suggesting multiple components are involved in Groucho competition with β-catenin. The variable ability of Groucho/TLE to oppose Wnt signaling in colon cancer cells suggests defects exist in one or more steps of the pathway.

## Competing interests

The authors declare that they have no competing interests.

## Authors' contributions

LA designed and carried out the protein interaction assays, reporter assays, and colon cancer cell transfections. KP created the LEF-1 amino acid substitution mutants and performed luciferase and western blot assays. MLW made contributions to the conception and design of the overall project. All authors made substantial contributions to the interpretation and critique of the data and each contributed to the drafting of the manuscript. All authors have read, edited, and approved the final manuscript.

## Pre-publication history

The pre-publication history for this paper can be accessed here:

http://www.biomedcentral.com/1471-2407/9/159/prepub
